# Impact of catheter ablation in asymptomatic patients with pre-excitation syndrome - real world evidence

**DOI:** 10.1007/s10840-026-02385-9

**Published:** 2026-07-28

**Authors:** Maximilian Moersdorf, Christian Massad, Mohammad Montaser Atasi, Yara Menassa, Han Feng, Michel Abou-Khalil, Alex El Darzi, Carlo El Khoury, Qussay Marashly, Rodolfo Montiel, Philipp Sommer, Amitabh C. Pandey, Christian Sohns, Nassir Marrouche

**Affiliations:** 1 Tulane Research Innovation for Arrhythmia Discovery, New Orleans, LA USA; 2 Department of Electrophysiology, Heart- and Diabetescenter NRW, Ruhr University Bochum, Medical School OWL (University Bielefeld), Bad Oeynhausen, Germany

**Keywords:** Pre-excitation syndrome, Heart failure, Catheter ablation, All-cause mortality

## Abstract

**Background:**

Pre-excitation syndromes such as Wolff–Parkinson–White increase risks of tachyarrhythmias and sudden cardiac death. While symptomatic patients undergo ablation, optimal management of asymptomatic individuals remains debated. This study aims to evaluate long-term outcomes of catheter ablation versus conservative management in asymptomatic patients utilizing real-world data.

**Methods:**

In this observational cohort study using the U.S. Collaborative Network in TriNetX, patients ≥ 12 to 50 years with asymptomatic pre-excitation between September 1, 2010 and October 1, 2024 were identified. After 1:1 propensity score matching, 5,852 patients were included. The primary endpoint was all-cause mortality. Secondary endpoints included hospitalization, syncope, new onset of heart failure (HF), ventricular tachycardia/fibrillation (VT/VF), atrial fibrillation (AF) and antiarrhythmic drug (AAD) use up to 10 years of follow-up. Outcomes were compared between patients undergoing ablation versus those receiving medical therapy (GDMT).

**Results:**

Over a mean follow-up of 1133 ± 1166 days, ablation was associated with reduced risks of the all-cause mortality (hazard ratio (HR) 0.25, 95%CI:0.13–0.48, *p* < 0.001), HF onset (HR 0.48, 95%CI:0.26–0.85, *p* = 0.011) and hospitalization rates (HR 0.47, 95%CI:0.37–0.60, *p* < 0.001). No significant differences were observed for new onset of VT/VF (HR 0.85, 95%CI:0.51–1.43, *p* = 0.534), AF (HR 1.61, 95%CI:1.00-2.62, *p* = 0.051) and syncope (HR 1.50, 95%CI:0.78–2.91, *p* = 0.224).

**Conclusion:**

In asymptomatic patients with pre-excitation, catheter ablation was associated with a reduced mortality, fewer hospitalizations, and HF onset. These findings support considering ablation as a proactive strategy in selected asymptomatic individuals.

**Graphical Abstract:**

**Figure:**
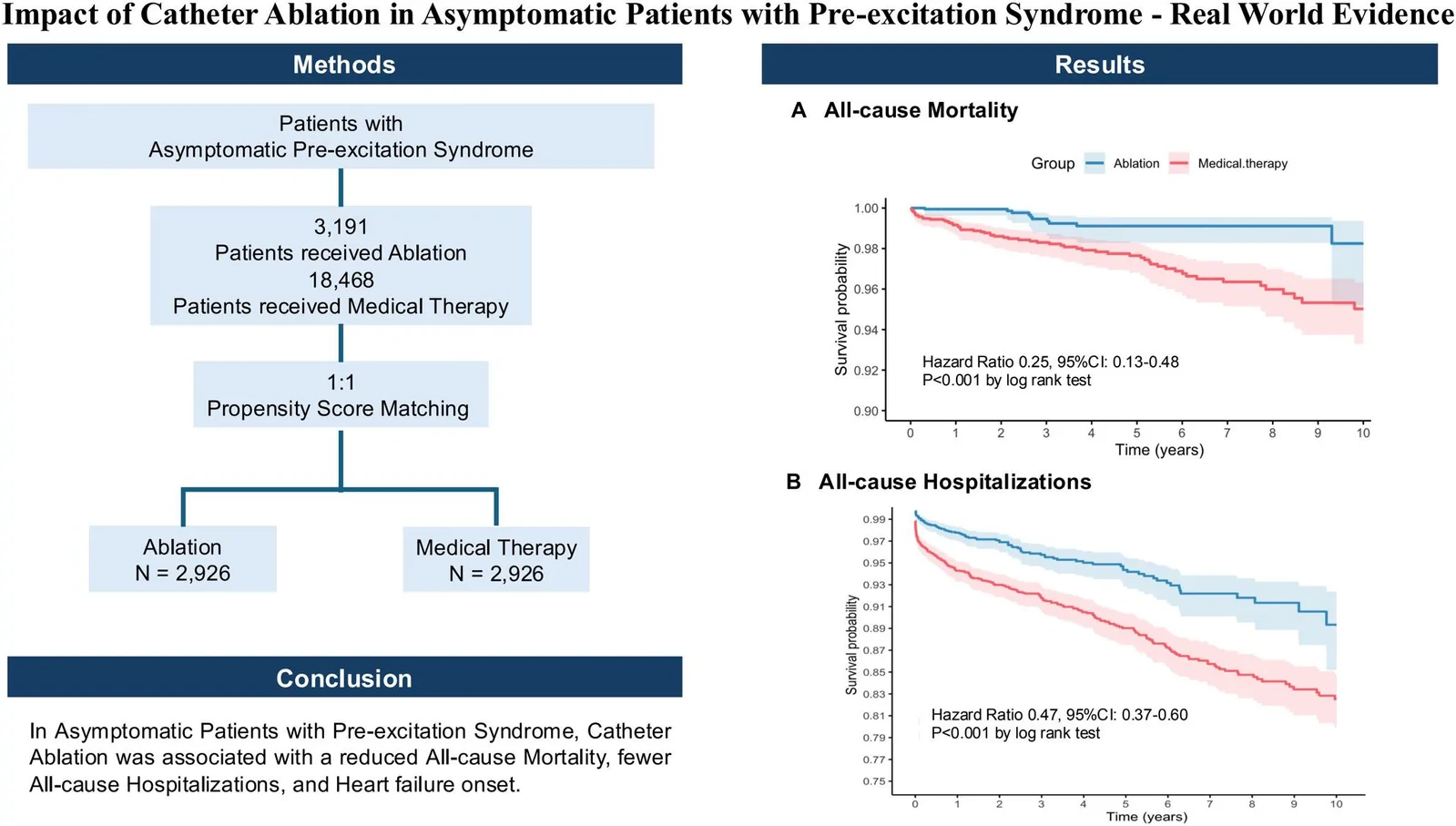
Impact of Catheter Ablation in Asymptomatic Patients with Pre-excitation Syndrome - Real World Evidence

**Supplementary Information:**

The online version contains supplementary material available at https://doi.org/10.1007/s10840-026-02385-9.

## Introduction

Ventricular pre-excitation due to an atrioventricular accessory pathway (AP), most associated with Wolff–Parkinson–White (WPW) pattern, is present in 0.1–0.3% of the general population. It carries a lifelong risk of supraventricular tachyarrhythmias, rapidly conducted atrial fibrillation (AF), and, in rare cases, sudden cardiac death (SCD) [1, 2]. While symptomatic individuals almost universally receive curative catheter ablation, the optimal management of asymptomatic patients remains debated because the absolute risk of life-threatening arrhythmias is considered to be low.

A substantial proportion of individuals remain asymptomatic throughout life, yet SCD or malignant ventricular arrhythmias may be the first manifestation of the condition [3]. The risk of SCD in asymptomatic ventricular pre-excitation is low but not negligible. A meta-analysis of 20 studies including 1,869 asymptomatic adults estimated an annual SCD incidence of approximately 0.13% per year, and pediatric cohorts suggest a similar or slightly higher risk in children and adolescents [4, 5]. Risk is higher in patients with high-risk pathway properties, including a shortest pre-excited RR interval during AF < 250 ms or an AP effective refractory period < 240 ms. Catheter ablation is highly effective for eliminating AP conduction, with contemporary series reporting acute success rates > 94% and low complication rates less than 1% [6, 7].

Early randomized and registry data, including the landmark prophylactic ablation trial by Pappone et al., demonstrated that ablation in asymptomatic WPW patients at a high risk for arrhythmias significantly reduces arrhythmic events compared with observation. However, these studies were relatively small, performed in high-volume centers with specific inclusion criteria, and often focused on young, highly selected patients.

The 2019 ESC Guidelines on supraventricular tachycardia recommend invasive risk stratification and consider prophylactic catheter ablation in asymptomatic patients with pre-excitation who have high-risk features, high-risk occupations (e.g., pilots, professional drivers), or competitive athletic participation [8]. The 2012 PACES/HRS expert consensus statement similarly suggests risk-stratified management in asymptomatic young patients and allows for prophylactic ablation in those with high-risk features or in whom SCD would be particularly catastrophic [9]. Recent narrative and practical reviews highlight the ongoing controversy and emphasize shared decision-making, given the tension between a low but real lifetime SCD risk and the small but finite procedural risk of ablation [10].

Despite these guidelines, real-world data describing long-term hard outcomes of catheter ablation versus conservative management in unselected, asymptomatic pre-excitation patients across a broad age spectrum are sparse [11]. In particular, the impact of ablation on mortality, heart failure (HF), AF, and health-care utilization in contemporary practice remains incompletely understood. This study addresses the evidence gap using a large, multicenter, propensity-matched cohort from 2010 to 2024.

## Methods

### Study design

This study utilized TriNetX, a global clinical research network that aggregates de-identified electronic medical records data from participating health care organizations [12]. The TriNetX database integrates data including patients’ demographics, diagnoses, procedures, medications, and laboratory results. All data provided through TriNetX network are de-identified and aggregated, ensuring compliance with the Health Insurance Portability and Accountability Act and other applicable US government regulations. Patient demographics and diagnoses were obtained according to the International Classification of Diseases (ICD), ninth and tenth versions (ICD-9 and ICD-10). Data on procedures, laboratory tests, and medications were also obtained using the TriNetX network. Further information about the TriNetX network is publicly available at https://trinetx.com/data-sets-analytics/.

### Patient population

Patients aged 12 to 50 years diagnosed with asymptomatic ventricular pre-excitation syndrome between September 1, 2010, and October 1, 2024, were identified. Pre-excitation syndrome was defined by ICD code I45.6. Patients with a prior diagnosis of palpitations (ICD code: R00.2), syncope and collapse (ICD code: R55), and cardiac arrest (ICD code: I46) were considered to be symptomatic and were excluded. Catheter ablation was identified using CPT code 93653. A complete list of all ICD-10-CM codes used in this analysis is provided in Supplemental Table 1.

Patients were stratified into ablation and conservative therapy cohorts. The index date was the date of ablation and in the conservative cohort, the time of pre-excitation diagnosis. Follow-up started the day after the ablation and continued until an endpoint occurred or 10 years elapsed.

### Outcomes

The primary outcome was all-cause mortality. Secondary endpoints included all-cause hospitalization, new onset of HF, VT/VF, syncope, AF or antiarrhythmic drug (AAD) use, as well as the need for a pacemaker or implantable defibrillator.

### Statistical analysis

All statistical analyses were conducted using the TriNetX online research platform. Chi-square analysis and independent sample t-tests were used to compare categorical variables and continuous variables, respectively. Patients were propensity-score matched in a 1:1 ratio based on demographic, clinical, medication, and laboratory covariates (Supplementary Table 2).

Cox regression analysis was used to estimate hazard ratios (HRs) with 95% confidence intervals (CIs) for the primary and secondary outcomes.

## Results

### Baseline characteristics

A total of 21,659 patients with asymptomatic pre-excitation syndrome were identified. 3,191 patients underwent catheter ablation, and 18,468 received conservative therapy. Before matching, ablation patients were younger (18.9 vs. 24.4; *p* < 0.001), more male sex (62.5% vs. 55.5%; *p* < 0.001), and had more comorbidities. Additional baseline characteristics before propensity score matching are provided in Supplementary Table 3.

After propensity matching, 5,852 patients remained evenly distributed between both groups (Supplementary Table 4). Baseline characteristics of the propensity score-matched cohorts are presented in Table 1. Mean QRS duration in the ablation cohort remained wider after matching, while it was only available in 16.8% of patients (116 vs. 104 milliseconds; *p* < 0.001). Mean corrected QT interval remained longer in the ablation group (442 vs. 432 milliseconds; *p* < 0.001) in the evaluated 11% of patients, too. Furthermore, mean heart rate (80 vs. 84 beats per minute; *p* < 0.001) and glomerular filtration rate (97 vs. 103 milliliter per minute; *p* < 0.001) were lower in the ablation group, while there was no difference in creatinine (1.1 vs. 0.8 milligram per deciliter; *p* = 0.159).

**Table 1:** Baseline characteristics after propensity score matching †

Characteristic	Ablation		Medical therapy		*P*- value	Standard difference
	(*n* = 2926)		(*n* = 2926)			
Age at Index — yr	19	± 9	19	± 10	0.697	0.010
Male sex — no. (%)	1820	(62)	1804	(62)	0.667	0.011
Body-mass index	25	± 7	25	± 8	0.406	0.029
Left Ventricular Ejection Fraction (LVEF) (%)	59	± 11	57	± 11	0.434	0.021
Atrial fibrillation and flutter	114	(4)	98	(3)	0.263	0.029
Persistent atrial fibrillation — no. (%)	10	(0.3)	10	(0.3)	1.000	< 0.001
QRS duration						
No. of patients evaluated (%)	489	(17)	495	(17)	0.834	0.005
Duration — ms	116	± 24	104	± 27	< 0.001	0.474
QTc interval						
No. of patients evaluated (%)	334	(11)	334	(11)	1.000	< 0.001
Duration — ms	442	± 34	432	± 33	< 0.001	0.309
Heart rate — bpm	80	± 17	84	± 20	< 0.001	0.212
Heart failure — no. (%)	46	(2)	59	(2)	0.201	0.034
Cardiomyopathy — no. (%)	52	(2)	65	(2)	0.225	0.032
Ischemic	10	(0.3)	10	(0.3)	1.000	< 0.001
Hypertensive diseases — no. (%)	142	(5)	166	(6)	0.160	0.037
COPD — no. (%)	10	(0.3)	10	(0.3)	1.000	< 0.001
Diabetes — %	47	(2)	48	(2)	0.918	0.003
Cerebral infarction — no. (%)	10	(0.3)	10	(0.3)	1.000	< 0.001
Presence of cardiac pacemaker — no. (%)	0	(0)	0	(0)	1.000	< 0.001
Presence of ICD — no. (%)	10	(0.3)	10	(0.3)	1.000	< 0.001
NT-pro BNP — pg/ml	1546	± 3122	2143	± 6218	0.597	0.121
Creatinine — mg/dl	1.1	± 6	0.8	± 1	0.159	0.062
Glomerular filtration rate (MDRD) — ml/min	97	± 25	103	± 32	< 0.001	0.209
Medication — no. (%)						
Beta blocker	536	(18)	555	(19)	0.524	0.017
Diuretics	99	(3)	120	(4)	0.148	0.038
ACE-inhibitor	81	(3)	97	(3)	0.223	0.032
Angiotensin II inhibitor	19	(1)	27	(1)	0.236	0.031
Spironolactone	17	(1)	25	(1)	0.215	0.032
Sacubitril	10	(0.3)	10	(0.3)	1.000	< 0.001
SGLT-2 inhibitor	10	(0.3)	10	(0.3)	1.000	< 0.001
Antiarrhythmics	657	(22)	724	(25)	0.024	0.059

†Plus–minus values are means ± SD. Body mass index is calculated as kilograms per square meter, Glomerular filtration rate is calculated as volume rate per 1.73 square meter. COPD denotes Chronic obstructive pulmonary disease, ICD implantable cardioverter defibrillator, ACE angiotensin-converting enzyme, SGLT-2 sodium-glucose transport protein 2 and SD standard deviation

### Primary endpoint

**Fig. 1:**
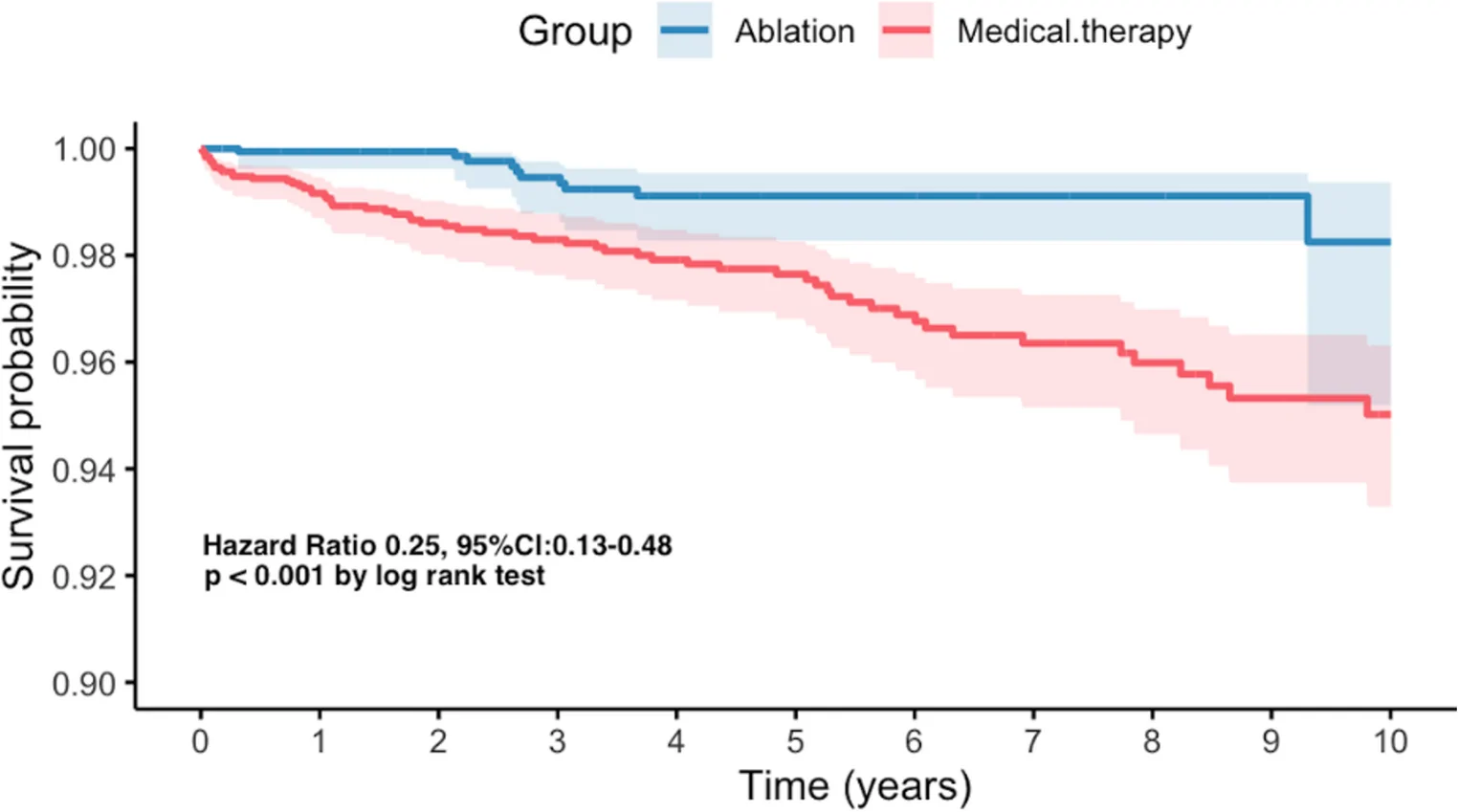
Incidence of primary endpoint events. Kaplan-Meier survival curve demonstrating risk of primary endpoint in patients with pre-excitation syndrome

Over a mean follow-up of 1133 ± 1166 days, early ablation was associated with a significantly lower incidence of mortality (≤ 10 vs. 61; HR 0.25, 95%CI:0.13–0.48, *p* < 0.001) (Fig. 1).

### Secondary outcomes

**Fig. 2:**
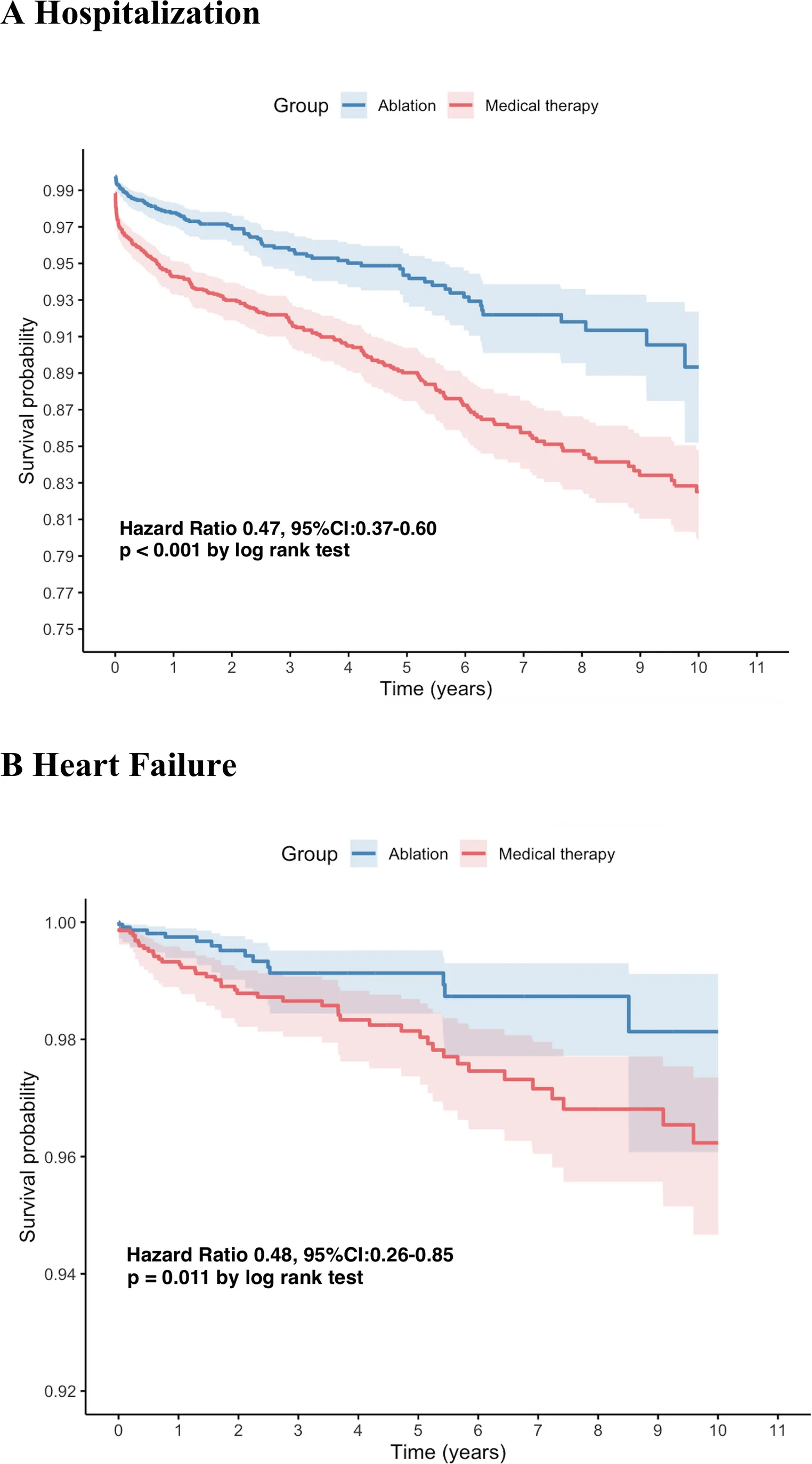
Incidence of hospitalization and heart failure. Kaplan-Meier survival curve demonstrating risk of hospitalization (**A**), new onset of heart failure (**B**)

Catheter ablation reduced all-cause hospitalization (HR 0.47, 95%CI:0.37–0.60, *p* < 0.001), new onset of HF (HR 0.48, 95%CI:0.26–0.85, *p* = 0.011), and lowers AAD Class I or III use (HR 0.81, 95%CI:0.73–0.91, *p* < 0.001) (Fig. 2).

**Fig. 3:**
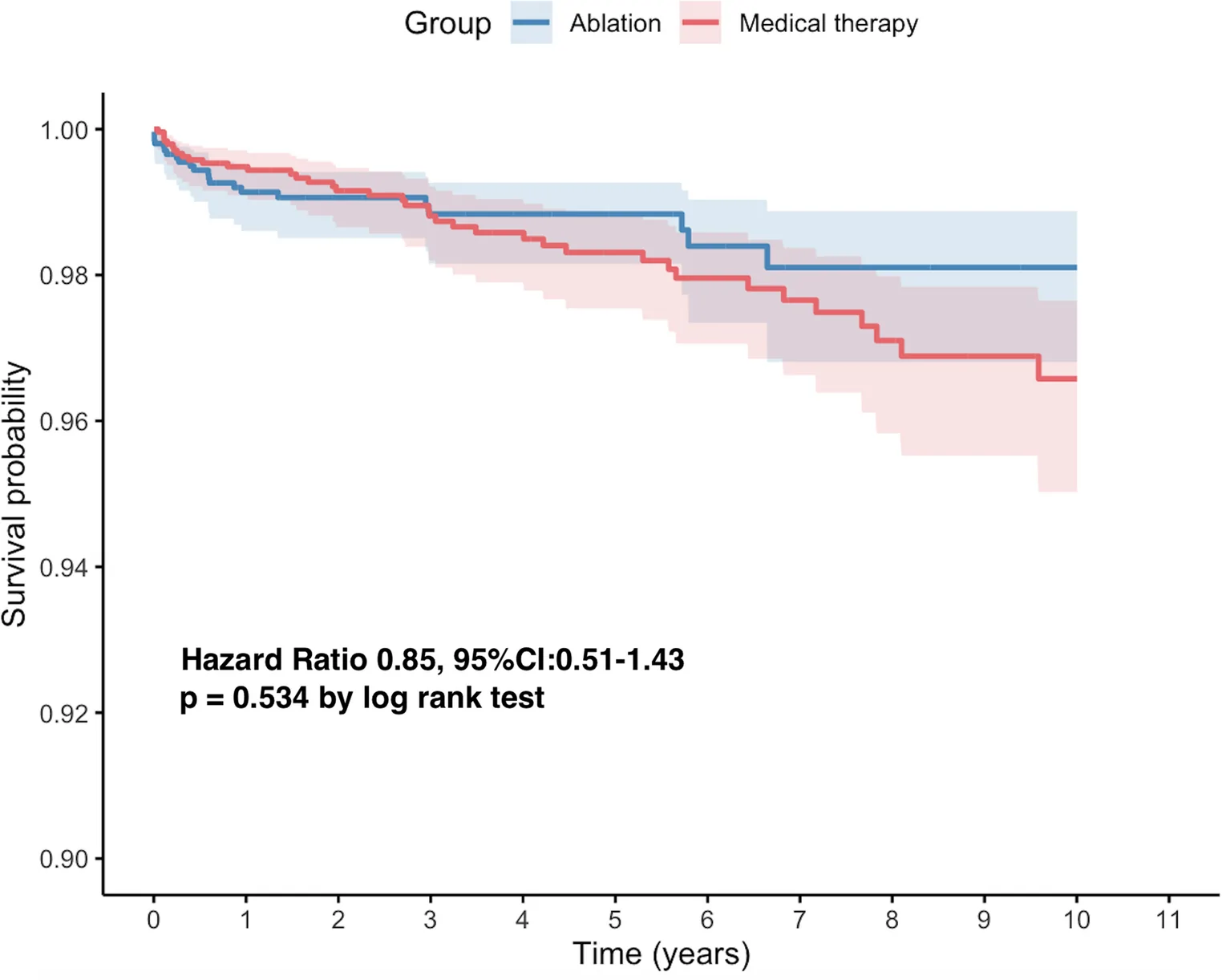
Incidence of ventricular tachycardia or fibrillation. Kaplan-Meier survival curve demonstrating ventricular tachycardia or fibrillation (VT/VF) rates

There were no significant differences in syncope rates (HR 1.50, 95%CI:0.78–2.91, *p* = 0.224) and occurrence of ventricular tachyarrhythmias including VT/VF (HR 0.85, 95%CI:0.51–1.43, *p* = 0.534; Fig. 3) or new onset of AF (HR 1.61, 95%CI:1.00–2.62, *p* = 0.051). The incidence of new pacemaker or implantable defibrillator implantation was numerically reduced after ablation (HR 0.54, 95%CI:0.27–1.08, *p* = 0.077).

## Discussion

In this large, real-world, propensity-matched cohort of asymptomatic individuals with ventricular pre-excitation, catheter ablation was associated with lower all-cause mortality, fewer hospitalizations, and a reduced incidence of new-onset HF. These findings provide strong and novel evidence that catheter ablation confers meaningful long-term clinical benefits even in patients who do not present with symptoms. However, ablation did not reduce the risk of malignant ventricular arrhythmias or syncope.

### Relationship to ESC and HRS guideline recommendations

The results reinforce and expand upon the 2019 ESC Supraventricular Tachycardia Guidelines, which recommend structured non-invasive evaluation and EPS for further risk stratification if uncertainty remains (Class IIa). The ESC further states that ablation is recommended in asymptomatic patients with high-risk EPS features (Class I) as well as in asymptomatic individuals with occupations that place them or others at risk should arrhythmias occur (Class I) [8]. However, the HRS/PACES 2012 consensus considers ablation reasonable when high-risk properties are identified (Class IIa), recommends ablation when syncope of suspected arrhythmic origin has occurred (Class I), and supports considering prophylactic ablation in young asymptomatic individuals (Class IIb) [9]. The significant reductions in mortality and HF events observed in our study suggest that the benefit of ablation may extend beyond the narrow subgroup defined by high-risk EPS parameters and may be relevant for a broader population of asymptomatic patients. Moreover, ablation procedures are associated with a very low complication rate of less than 1% [13].

### Occurrence of arrhythmia

Early randomized data from Pappone et al. demonstrated that prophylactic ablation in asymptomatic WPW patients at high risk for arrhythmias reduced new onset AF and malignant arrhythmias [14]. A following larger registry study included WPW syndromes regardless of their symptoms or risk and found that life-threatening arrhythmias such as pre-excited AF with rapid conduction occurred predominantly in non-ablated patients [13]. Whereas other data described no difference in AF by undergoing ablation [2, 15]. Our data did not find a significant reduction of VT/VF or AF in the ablation group, which might be due to a lower risk population. However, there was a trend to a higher AF onset in the ablation group (35 vs. 32 patients, *p* = 0.051).

Several pediatric cohort studies, such as Santinelli et al., revealed that even asymptomatic children may harbor rapidly conducting APs, challenging the assumption that asymptomatology equates to low risk [5]. Furthermore, Di Pino et al. and Brembilla-Perrot et al. showed that electrophysiological high-risk markers often appear in asymptomatic patients and cannot be reliably predicted by clinical presentation alone [16–18].

### Hospitalization and heart failure outcomes

One of the most notable findings of our study is the significant reduction in all-cause hospitalization among ablated patients. This aligns with population-based studies demonstrating elevated healthcare utilization among individuals with pre-excitation due to arrhythmic events, anxiety-driven emergency evaluations, and development of arrhythmias or HF [2, 13, 19].

Even more importantly, we observed a substantial reduction in new-onset HF among ablated patients. While HF has historically been under-recognized in the WPW population, several mechanisms support this association, such as tachycardia-mediated cardiomyopathy due to recurrent supraventricular tachycardia or intermittent rapid pre-excited conduction, which may lead to progressive ventricular dysfunction. Furthermore, intermittent AF or atrial tachycardia episodes can promote atrial remodeling with fibrosis, impairing diastolic function. Additionally, dyssynchronous activation according to persistent pre-excitation may lead to abnormal mechanical activation patterns, especially in patients with right-sided pathways. Evidence supporting these mechanisms includes observations from pediatric cohorts and adult registries showing increased AF and HF in non-ablated WPW patients [19, 20]. In case of abnormal conduction-induced cardiomyopathy, the decreased left ventricular function could even recover in young children after ablation of the AP [21]. Our data now provide large-scale confirmation that ablation reduces HF onset—suggesting meaningful myocardial protection over time, even in previously asymptomatic patients.

### Mortality

In addition to the reduction in HF development, our findings may also help explain the observed mortality benefit in the ablation group. Once HF develops, even in relatively young or structurally normal patients, mortality rises substantially. Contemporary population studies indicate that new-onset HF is associated with a 5-year mortality approaching 40–50%, independent of initial ejection fraction or etiology [22, 23]. Because arrhythmia-mediated or pre-excitation–related hemodynamic stress may predispose to progressive myocardial dysfunction, preventing the onset of HF through early pathway elimination may interrupt a downstream clinical trajectory strongly linked to premature mortality. Thus, the lower mortality observed in the ablation cohort is consistent with the known prognostic implications of avoiding new-onset HF. Besides lower HF onset in the ablation group, reduced mortality might be explained by a decrease in SCD.

Meta-analyses estimated annual SCD risks of 0.07–0.45% in asymptomatic pre-excitation [4]. While this risk appears low annually, cumulative exposure over decades can substantially increase lifetime SCD risk, especially in young patients. Ablation of AP reduces the occurrence of malignant arrhythmias and the associated SCD. Furthermore, SCD is likely underestimated in all-cause mortality analyses due to misclassification of out-of-hospital deaths and the low absolute but lifelong cumulative risk. The observed mortality reduction after AP ablation may therefore reflect prevention of arrhythmic deaths not fully captured in administrative datasets. These insights support the idea that asymptomatic WPW is not a uniformly benign condition.

### Novel insights from the present study

Our study is the first large-scale analysis to demonstrate that catheter ablation is associated with a 75% reduction in all-cause mortality, a 52% reduction in new-onset HF, and a marked reduction in all-cause hospitalization.

These endpoints extend beyond arrhythmia prevention and suggest a deeper, long-term physiological benefit of eliminating pre-excitation. Potential mechanisms include avoidance of intermittent rapid pre-excited AF episodes, prevention of tachycardia-mediated cardiomyopathy, and reduced atrial remodeling. Nonetheless, these findings should be interpreted cautiously given the observational design and the potential for residual confounding. The observed mortality benefit should be considered hypothesis-generating and warrant confirmation in prospective randomized studies.

### Limitations

This is a retrospective observational study, therefore susceptible to selection bias and unmeasured confounders, which restrict the ability to demonstrate direct causality. Despite propensity score matching, several variables including QRS duration (116 vs. 104 ms), QTc interval (442 vs. 432 ms), heart rate (80 vs. 84 bpm), and glomerular filtration rate (97 vs. 103 ml/min) remained imbalanced between groups. In particular, the wider QRS duration in the ablation cohort may reflect more manifest pre-excitation and a higher-risk phenotype, which could have contributed to residual confounding and influenced outcome differences. Data were derived from the U.S. TriNetX network and dependent on coding accuracy across participating institutions, which may have introduced additional heterogeneity. The definition of asymptomatic pre-excitation relied on the absence of administrative coding for palpitations, syncope, and cardiac arrest. Therefore, misclassification due to incomplete or inaccurate coding cannot be excluded. In addition, differences in healthcare access and treatment availability among patients may have influenced outcomes.

Further details, including pathway localization, the appearance of multiple pathways, operator/center volume, and procedural specifics such as the ablation energy source, were not available. In addition, pathways located close to the conduction system may have been managed more conservatively, potentially contributing to residual selection bias and limiting generalizability. The TriNetX database does not provide detailed procedural information regarding acute procedural success, long-term ablation durability, results of electrophysiological testing without ablation or non-invasive evaluations. Procedural complications were not consistently available within the TriNetX network. Therefore, these clinically relevant variables could not be assessed in the present study. Nevertheless, contemporary studies have reported high acute success rates and low complication rates for AP ablation procedures. The timing of ablation may also reflect unmeasured biases (healthcare access, referral patterns). Finally, the cause-specific mortality was not available.

Despite these limitations, the large multicenter dataset strengthens the validity of our findings.

## Conclusion

In this large real-world cohort of asymptomatic patients with pre-excitation, catheter ablation was associated with a reduced mortality, fewer hospitalizations, and HF onset. These findings indicate the necessity for further studies on prophylactic ablation as a proactive strategy in selected asymptomatic individuals.

## Supplementary Information

Below is the link to the electronic supplementary material.

Supplementary File 1 (DOCX 23.1 KB)

## Data Availability

This study utilized TriNetX, a global clinical research network that aggregates de-identified electronic medical records data from participating health care organizations (12). The TriNetX database integrates data including patients’ demographics, diagnoses, procedures, medications, and laboratory results. All data provided through TriNetX network are de-identified and aggregated, ensuring compliance with the Health Insurance Portability and Accountability Act and other applicable US government regulations. Patient demographics and diagnoses were obtained according to the International Classification of Diseases (ICD), ninth and tenth versions (ICD-9 and ICD-10). Data on procedures, laboratory tests, and medications were also obtained using the TriNetX network. Further information about the TriNetX network is publicly available at https://trinetx.com/data-sets-analytics/.

## Abbreviations

AAD: Antiarrhythmic drug
AF: Atrial fibrillation
AP: Accessory pathway
CI: Confidence interval
HF: Heart failure
HR: Hazard ratio
ICD: International classification of diseases
SCD: Sudden cardiac death
VT/VF: Ventricular tachycardia/fibrillation
WPW: Wolff–Parkinson–White
