# Supplementary material for: Impact of catheter ablation in asymptomatic patients with pre-excitation syndrome - real world evidence
Source: J Interv Card Electrophysiol. 2026 Jul 28;69(8):2557–65. doi: 10.1007/s10840-026-02385-9 (PMC13739948; doi:10.1007/s10840-026-02385-9)
Supplement: Supplementary file 1 [file 10840_2026_2385_MOESM1_ESM.docx]

## Supplemental Material

**Supplementary Table 1.** ICD-10-CM codes used for diagnosis of comorbidities

| Pre-excitation syndrome | I45.6 |
| --- | --- |
| Cardiac arrest | I46 |
| Syncope and collapse | R55 |
| Palpitation | R00.2 |
| Heart failure | I50 |
| Ventricular tachycardia | I47.2 |
| Ventricular fibrillation and flutter | I49.0 |
| Atrial Fibrillation and flutter | I48 |
| Atrial Fibrillation | I48.0 |
|  | I48.1 |
|  | I48.2 |
|  | I48.91 |
| Essential hypertension | I10 |
| Diabetes mellitus | E08-E13 |
| Other chronic obstructive pulmonary disease | J44 |
| Cerebral infarction | I63 |
| Cardiomyopathy | I42 |
| Dilated cardiomyopathy | I42.0 |
| Ischemic cardiomyopathy | I25.5 |
| Presence of cardiac pacemaker | Z95.0 |
| Presence of automatic (implantable) cardiac defibrillator | Z95.810 |

**Supplementary Table 2.** Variables included in propensity score

| **Demographics** | Age at Index |
| --- | --- |
|  | Sex |
| **Diagnosis** | Essential hypertension |
|  | Diabetes mellitus |
|  | Other chronic obstructive pulmonary disease |
|  | Atrial fibrillation and flutter |
|  | Persistent atrial fibrillation |
|  | Cardiomyopathy |
|  | Dilated cardiomyopathy |
|  | Ischemic cardiomyopathy |
|  | Cerebral infarction |
|  | Heart failure |
|  | Presence of cardiac pacemaker |
|  | Presence of automatic (implantable) cardiac defibrillator |
| **Medications** | Beta blocker |
|  | Diuretics |
|  | ACE-inhibitor |
|  | Angiotensin II inhibitor |
|  | Sacubitril |
|  | Spironolactone |
|  | Sodium-glucose co-transporter 2 (SGLT2) inhibitors |
|  | Antiarrhythmics |
| **Laboratory parameters** | Body-mass index |
|  | Body surface area |
|  | Heart rate — bpm |
|  | QRS — ms |
|  | Corrected QT interval (QTc) — ms |
|  | Creatinine [Mass/volume] in Serum, Plasma or Blood |
|  | Glomerular filtration rate/1.73 sq M.predicted [Volume Rate/Area] in Serum, Plasma or Blood by Creatinine-based formula (MDRD) |
|  | Hemoglobin [Mass/volume] |
|  | Natriuretic peptide.B prohormone N-Terminal [Mass/volume] in Serum, Plasma or Blood |
|  | Left Ventricular Ejection Fraction (LVEF) (%) |

**Supplementary Table 3.** Baseline characteristics before propensity score matching- extended†

| **Characteristic** |  | **Ablation** | |  | **Medical therapy** | |  | **P- value** |  | **Standard difference** |
| --- | --- | --- | --- | --- | --- | --- | --- | --- | --- | --- |
|  |  | **(n=3191)** | |  | **(n=18468)** | |  |  |  |  |
| **Demographics** |  |  |  |  |  |  |  |  |  |  |
| Age at Index— yr |  | 19 | ±9 |  | 24 | ±11 |  | <0.001 |  | 0.544 |
| Female sex — no. (%) |  | 1194 | (37) |  | 10241 | (55) |  | <0.001 |  | 0.144 |
| Male sex — no. (%) |  | 1994 | (62) |  | 8214 | (44) |  | <0.001 |  | 0.143 |
| Unknown sex — no. (%) |  | 10 | (.3) |  | 13 | (.1) |  | <0.001 |  | 0.056 |
| **Comorbidities — no. (%)** |  |  |  |  |  |  |  |  |  |  |
| Essential hypertension |  | 156 | (5) |  | 881 | (5) |  | 0.773 |  | 0.006 |
| Diabetes mellitus |  | 49 | (2) |  | 310 | (2) |  | 0.559 |  | 0.011 |
| COPD |  | 10 | () |  | 46 | () |  | 0.509 |  | 0.012 |
| Cardiomyopathy |  | 62 | (2) |  | 199 | (1) |  | <0.001 |  | 0.071 |
| Dilated cardiomyopathy |  | 10 | () |  | 25 | () |  | 0.021 |  | 0.038 |
| Ischemic cardiomyopathy | | 10 | () |  | 10 | () |  | <0.001 |  | 0.061 |
| Heart failure |  | 54 | (2) |  | 168 | (1) |  | <0.001 |  | 0.069 |
| Atrial fibrillation and flutter | | 145 | (5) |  | 169 | (1) |  | <0.001 |  | 0.224 |
| Persistent atrial fibrillation | | 10 | () |  | 10 | () |  | <0.001 |  | 0.061 |
| Presence of cardiac pacemaker | | 0 | () |  | 17 | () |  | 0.086 |  | 0.043 |
| Presence of ICD |  | 10 | () |  | 10 | () |  | <0.001 |  | 0.061 |
| Cerebral infarction |  | 10 | () |  | 65 | () |  | 0.732 |  | 0.007 |
| **Medication — no. (%)** |  |  |  |  |  |  |  |  |  |  |
| Beta blocker |  | 683 | (21) |  | 1282 | (7) |  | <0.001 |  | 0.424 |
| Diuretics |  | 106 | (3) |  | 642 | (3) |  | 0.659 |  | 0.009 |
| ACE-inhibitor |  | 83 | (3) |  | 463 | (3) |  | 0.754 |  | 0.006 |
| Angiotensin II inhibitor |  | 77 | (2) |  | 319 | (2) |  | 0.008 |  | 0.048 |
| Spironolactone |  | 18 | (1) |  | 149 | (1) |  | 0.147 |  | 0.029 |
| Sacubitril |  | 10 | () |  | 16 | () |  | 0.001 |  | 0.051 |
| SGLT-2 inhibitors |  | 10 | () |  | 19 | () |  | 0.003 |  | 0.046 |
| Antiarrhythmics |  | 737 | (23) |  | 2498 | (14) |  | <0.001 |  | 0.249 |
| **Laboratory parameters** |  |  |  |  |  |  |  |  |  |  |
| Body-mass index |  | 25 | ±7 |  | 27 | ±8 |  | <0.001 |  | 0.281 |
| Body surface area |  | 2 | ±.5 |  | 2 | ±.5 |  | 0.064 |  | 0.118 |
| Heart rate — bpm |  | 80 | ±17 |  | 83 | ±18 |  | <0.001 |  | 0.163 |
| Creatinine — mg/dl |  | 1 | ±6 |  | 1 | ±2 |  | 0.049 |  | 0.047 |
| GFR (MDRD) — ml/min |  | 97 | ±25 |  | 100 | ±30 |  | 0.019 |  | 0.110 |
| NT-pro BNP — pg/ml |  | 1308 | ±2704 |  | 1594 | ±5109 |  | 0.704 |  | 0.070 |
| LVEF (%) |  | 58 | ±10 |  | 57 | ±11 |  | 0.395 |  | 0.108 |
| QRS duration — ms |  | 116 | ±25 |  | 110 | ±26 |  | <0.001 |  | 0.534 |
| Corrected QT interval — ms | | 441 | ±34 |  | 431 | ±32 |  | <0.001 |  | 0.312 |

†Plus–minus values are means ±SD. Body mass index is calculated as kilograms per square meter, Glomerular filtration rate (GFR) is calculated as volume rate per 1.73 square meter. COPD denotes Chronic obstructive pulmonary disease, ICD implantable cardioverter defibrillator, ACE angiotensin-converting enzyme, SGLT-2 sodium-glucose transport protein 2, LVEF left ventricular ejection fraction and SD standard deviation.

**Supplementary Table 4**. Baseline characteristics after propensity score matching – extended†

| **Characteristic** |  | **Ablation** | |  | **Medical therapy** | |  | **P- value** |  | **Standard difference** |
| --- | --- | --- | --- | --- | --- | --- | --- | --- | --- | --- |
|  |  | **(n=2926)** | |  | **(n=2926)** | |  |  |  |  |
| **Demographics** |  |  |  |  |  |  |  |  |  |  |
| Age at Index— yr |  | 19 | ±9 |  | 19 | ±10 |  | 0.697 |  | 0.010 |
| Female sex — no. (%) |  | 1103 | (38) |  | 1119 | (38) |  | 0.667 |  | 0.011 |
| Male sex — no. (%) |  | 1820 | (62) |  | 1804 | (62) |  | 0.667 |  | 0.011 |
| Unknown sex — no. (%) |  | 10 | (.3) |  | 10 | (.3) |  | 1.000 |  | 0.000 |
| **Comorbidities — no. (%)** |  |  |  |  |  |  |  |  |  |  |
| Essential hypertension |  | 142 | (5) |  | 166 | (6) |  | 0.160 |  | 0.037 |
| Diabetes mellitus |  | 47 | (2) |  | 48 | (2) |  | 0.918 |  | 0.003 |
| COPD |  | 10 | () |  | 10 | () |  | 1.000 |  | 0.000 |
| Cardiomyopathy |  | 52 | (2) |  | 65 | (2) |  | 0.225 |  | 0.032 |
| Dilated cardiomyopathy |  | 10 | () |  | 12 | () |  | 0.669 |  | 0.011 |
| Ischemic cardiomyopathy | | 10 | () |  | 10 | () |  | 1.000 |  | 0.000 |
| Heart failure |  | 46 | (2) |  | 59 | (2) |  | 0.200 |  | 0.033 |
| Atrial fibrillation and flutter | | 114 | (4) |  | 98 | (3) |  | 0.263 |  | 0.029 |
| Persistent atrial fibrillation | | 10 | () |  | 10 | () |  | 1.000 |  | 0.000 |
| Presence of cardiac pacemaker | | 0 | () |  | 0 | () |  | 1.000 |  | 0.000 |
| Presence of ICD |  | 10 | () |  | 10 | () |  | 1.000 |  | 0.000 |
| Cerebral infarction |  | 10 | () |  | 10 | () |  | 1.000 |  | 0.000 |
| **Medication — no. (%)** |  |  |  |  |  |  |  |  |  |  |
| Beta blocker |  | 536 | (18) |  | 555 | (19) |  | 0.524 |  | 0.017 |
| Diuretics |  | 99 | (3) |  | 120 | (4) |  | 0.148 |  | 0.038 |
| ACE-inhibitor |  | 81 | (3) |  | 97 | (3) |  | 0.223 |  | 0.032 |
| Angiotensin II inhibitor |  | 19 | (1) |  | 27 | (1) |  | 0.236 |  | 0.031 |
| Spironolactone |  | 17 | (1) |  | 25 | (1) |  | 0.215 |  | 0.032 |
| Sacubitril |  | 10 | () |  | 10 | () |  | 1.000 |  | <0.001 |
| SGLT-2 inhibitors |  | 10 | () |  | 10 | () |  | 1.000 |  | <0.001 |
| Antiarrhythmics |  | 657 | (22) |  | 724 | (25) |  | 0.024 |  | 0.059 |
| **Laboratory parameters** |  |  |  |  |  |  |  |  |  |  |
| Body-mass index |  | 25 | ±7 |  | 25 | ±8 |  | 0.406 |  | 0.029 |
| Body surface area |  | 2 | ±.5 |  | 2 | ±.5 |  | 0.684 |  | 0.011 |
| Heart rate — bpm |  | 80 | ±17 |  | 84 | ±20 |  | <0.001 |  | 0.212 |
| Creatinine — mg/dl |  | 1.1 | ±6 |  | 0.8 | ±1 |  | 0.159 |  | 0.062 |
| GFR (MDRD) — ml/min |  | 97 | ±25 |  | 103 | ±32 |  | <0.001 |  | 0.209 |
| NT-pro BNP — pg/ml |  | 1546 | ±3122 |  | 2143 | ±6218 |  | 0.597 |  | 0.121 |
| LVEF (%) |  | 59 | ±11 |  | 57 | ±11 |  | 0.434 |  | 0.021 |
| QRS duration — ms |  | 116 | ±24 |  | 104 | ±27 |  | <0.001 |  | 0.474 |
| Corrected QT interval — ms | | 442 | ±34 |  | 432 | ±32.6 |  | <0.001 |  | 0.309 |

†Plus–minus values are means ±SD. Body mass index is calculated as kilograms per square meter, Glomerular filtration rate (GFR) is calculated as volume rate per 1.73 square meter. COPD denotes Chronic obstructive pulmonary disease, ICD implantable cardioverter defibrillator, ACE angiotensin-converting enzyme, SGLT-2 sodium-glucose transport protein 2, LVEF left ventricular ejection fraction and SD standard deviation.
